# Estimating Indoor PM_2.5_ and CO Concentrations in Households in Southern Nepal: The Nepal Cookstove Intervention Trials

**DOI:** 10.1371/journal.pone.0157984

**Published:** 2016-07-07

**Authors:** Chen Chen, Scott Zeger, Patrick Breysse, Joanne Katz, William Checkley, Frank C. Curriero, James M. Tielsch

**Affiliations:** 1 Department of Environmental Health Sciences, Johns Hopkins Bloomberg School of Public Health, Baltimore, MD, United States of America; 2 Department of Biostatistics, Johns Hopkins Bloomberg School of Public Health, Baltimore, MD, United States of America; 3 Department of International Health, Johns Hopkins Bloomberg School of Public Health, Baltimore, MD, United States of America; 4 Division of Pulmonary and Critical Care, Department of Medicine, Johns Hopkins School of Medicine, Baltimore, MD, United States of America; 5 Department of Epidemiology, Johns Hopkins Bloomberg School of Public Health, Baltimore, MD, United States of America; 6 Department of Global Health, Milken Institute School of Public Health, George Washington University, Washington, DC, United States of America; University of Rochester Medical Center, UNITED STATES

## Abstract

High concentrations of household air pollution (HAP) due to biomass fuel usage with unvented, insufficient combustion devices are thought to be an important health risk factor in South Asia population. To better characterize the indoor concentrations of particulate matter (PM_2.5)_ and carbon monoxide (CO), and to understand their impact on health in rural southern Nepal, this study analyzed daily monitoring data collected with DataRAM pDR-1000 and LASCAR CO data logger in 2980 households using traditional biomass cookstove indoor through the Nepal Cookstove Intervention Trial–Phase I between March 2010 and October 2011. Daily average PM_2.5_ and CO concentrations collected in area near stove were 1,376 (95% CI, 1,331–1,423) μg/m^3^ and 10.9 (10.5–11.3) parts per million (ppm) among households with traditional cookstoves. The 95^th^ percentile, hours above 100μg/m^3^ for PM_2.5_ or 6ppm for CO, and hours above 1000μg/m^3^ for PM_2.5_ or 9ppm for CO were also reported. An algorithm was developed to differentiate stove-influenced (SI) periods from non-stove-influenced (non-SI) periods in monitoring data. Average stove-influenced concentrations were 3,469 (3,350–3,588) μg/m^3^ for PM_2.5_ and 21.8 (21.1–22.6) ppm for CO. Dry season significantly increased PM_2.5_ concentration in all metrics; wood was the cleanest fuel for PM_2.5_ and CO, while adding dung into the fuel increased concentrations of both pollutants. For studies in rural southern Nepal, CO concentration is not a viable surrogate for PM_2.5_ concentrations based on the low correlation between these measures. In sum, this study filled a gap in knowledge on HAP in rural Nepal using traditional cookstoves and revealed very high concentrations in these households.

## Introduction

Approximately 3 billion people worldwide rely on solid fuels (biomass or coal) for cooking and heating due to lack of access to cleaner fuels [1,2]. Solid fuels are typically used with unvented, inefficient combustion devices leading to high emissions of toxic pollutants due to incomplete combustion, including two main pollutants contributing to morbidity and mortality: particulate matter (PM) of various sizes, and carbon monoxide (CO) [2,3]. High concentration of fine PM is a known risk factor for cardiopulmonary adverse outcomes. CO is associated with fatality and acute exposure-related reduction of exercise tolerance and also a marker for PM exposure in some studies [3,4]. Combining exposure to all related pollutants, household air pollution (HAP) due to solid fuels was estimated to account for 3.5 million deaths across the world in 2010, and was the leading risk factor for death in South Asia [5]. To reduce the disease burden due to HAP in households using solid fuels, understanding the exposure-outcome relationship is critical [3,6].

Difference in stove design, fuels used and cooking practices across regions can lead to large variability in HAP concentrations. Therefore information is needed to assess exposures across different study locations [6]. Research on solid fuel related pollutants and health impacts are limited in low income countries like Nepal [7]. Previous studies documented high HAP concentrations during cooking in Nepalese houses using biomass fuel: 4,741 μg/m^3^ and 13.7 parts per million (ppm) for PM_2.5_ and CO, respectively [8,9]. In rural India, recent studies found 24-hour average concentrations of 686 μg/m^3^ for PM_2.5_ and 2.6 ppm for CO among households using biomass fuel [10], and 48-hour average concentrations of 1,250 μg/m^3^ for PM_2.5_ and 10.8 ppm for CO in households using traditional cookstoves [11]. These levels are many times higher than current air quality guidelines published by the World Health Organization (WHO): 25 μg/m^3^ for 24-hour average ambient PM_2.5_ exposure and 6 ppm for 24-hour average indoor CO exposure [4,12].

The Nepal Cookstove Intervention Trial–Phase I (NCIT-I) was designed to assess and reduce adverse health effects (mainly acute lower respiratory infection) of biomass fuel smoke exposure among women and young children with installation of enhanced, ventilated biomass stoves to replace the traditional open burning mud stoves. Continuous daily concentrations of PM_2.5_ and CO were measured before and after the installation of new stove in area close to stove among eligible households. This paper reports the methods for quantifying daily indoor PM_2.5_ and CO concentrations using monitoring data collected before enhanced stove installation in preparation for further analysis of related health outcomes. Issues and systematic solutions regarding data reduction and data analysis for daily continuous HAP concentrations are detailed as well as method for determining the pollutant concentration during cooking or stove-influenced (SI) times.

## Methods

### Data Collection

NCTI-I was conducted in Sarlahi, a district on Nepal’s southern border with Bihar State in India. Residents of all households in four Village Development Committees were screened for enrollment eligibility. The final eligible households only used traditional biomass cookstove indoor and had a married woman aged 15–30 or a child younger than 36 months. Detailed methods for study design and enrollment criteria have been published previously [13]. Between March 2010 and July 2012, all participating households in NCIT-I received two HAP assessments, once before the new stove was installed and once afterwards. This assessment comprised measurement of PM_2.5_ and CO concentrations, temperature, and relative humidity every 10 seconds for a period of approximately 21 hours. For each household-day, measurements started at approximately 3:00 pm and stopped around 12:00 pm the following day. This interval covered nearly all the cooking events since lunch is not a typical meal in rural Nepal. In addition, household characteristics including roof and wall material, room dimensions, and number of external openings in the kitchen were collected during enrollment. Date (season) and fuel type (wood, animal dung, crop waste) were collected during environmental sampling.

HAP concentrations were measured using a package of instruments including the DataRAM pDR-1000 (Thermo Scientific, Franklin, MA), the LASCAR CO data logger (EL-USB-CO300, Erie, PA), and the HOBO U10 Temperature and Humidity (TH) Data Logger (Onset Computer Corporation, Pocasset MA), all recording data in 10-second intervals. A package of instruments was placed approximately 1 meter in front of the stove and approximately 1.5 meters off the floor during each measurement to best capture the exposure to individuals who were cooking.

This study was approved by the institutional review boards (IRB) of the Johns Hopkins Bloomberg School of Public Health and the Institute of Medicine, Tribhuvan University, Kathmandu, Nepal.

As a significant proportion of this population was illiterate, verbal informed consent was received from all participating households and individuals and consent was documented directly on data collection forms and entered into the study database. All IRBs approved the consent procedures and all other procedures used in these studies. The trials are registered at Clinicaltrials.gov (NCT 00786877).

### Pre-Processing HAP Signals

To ensure consistency of quality control for data collected in NCIT-I, all daily pollution records collected before and after stove installation were pre-processed together; a total of 7684 PM and 6615 CO measurements. Measurements of PM concentration were removed from analysis when a) data were in the wrong format or could not be connected to an eligible household (2.6%), b) total sampling time was shorter than 18 hours (12.2%) and c) PM data with abrupt change (>5%) from baseline during sampling, or with an entirely flat line during sampling while cooking-time peaks were observed in corresponding CO results, were identified as physically implausible and a result of machine malfunction (1.6%). Similar quality control was conducted for CO measurements (3.6%, 19.7%, 3.3% removed from analysis respectively). The pDR-1000 is a passive nephelometric device and measured PM concentrations can be biased due to variations in ambient humidity and particulate matter composition. PM concentrations were adjusted to account for these factors using an algorithm described previously, resulting in gravimetric equivalent PM_2.5_ values [14]. PM_2.5_ measurements that lacked concurrent temperature and humidity measurements necessary for adjustment were removed (3.4%). Correspondingly, 10.7% CO measurements were removed due to missing PM_2.5_ measurements.

Drift of all PM_2.5_ measurements was calculated by subtracting the machine reported internal average concentration from the manually calculated unadjusted time-weighted average concentration of real-time pDR readings [15,16]. In this project, given the high average concentration for PM_2.5_, the ratio of drift over daily average PM_2.5_ concentration worked better as an exclusion criterion than the absolute value. Measurements with a drift ratio higher than 50% were removed (0.5%). After eliminating these data, the mean drift ratio was 1.3% and the greatest drift was 262 μg/m^3^, both acceptable when compared with the unadjusted time-weighted average concentration of 1712 μg/m^3^. Since the drift might have occurred at any point after the start of sampling and the drift was proportionally small, no further drift adjustment was performed.

The measurement range for pDR-1000 is 1 to 400,000 μg/m^3^, with 1 μg/m^3^ resolution; PM_2.5_ concentrations below the limit of detection were recorded as 0 μg/m^3^ [17]. To account for possible bias caused by this setting, all 0 μg/m^3^ PM_2.5_ measurements were adjusted to a value that is closest to half of the limit of detection 0.5 μg/m^3^, and is equal to or higher than the 1 μg/m^3^ resolution (1 μg/m^3^ in this case). Similarly, since the limit of detection for the LASCAR CO data logger was calculated to be 1.1 ppm, with 0.5 ppm resolution, all 0 ppm CO measurements were adjusted to the resolution (0.5 ppm in this case). 11.3% of PM measurements and 16.1% CO measurements were replaced.

To ensure that concentration excursions shorter than 30 seconds would be discounted, a running median of length 5 was applied to the 10-second data before aggregation into data with 5-minute intervals. These excursions may have been caused by unexpected disturbances to the sampling machine, for example by being bumped. Aggregating the filtered 10-second data into averages of 5 minutes reduced the size of the dataset while preserving the original diurnal trends in concentration.

### Quantifying daily Average HAP

Daily average concentration for each pollutant measure was estimated by the arithmetic mean of the observed 5-minute interval values derived from the smoothed 10-second time series. In addition to daily averages, three additional metrics were calculated for each household to more fully summarize distributions of PM_2.5_ and CO concentrations during each daily measurement period: the 95^th^ percentile, hours above 100 μg/m^3^ for PM_2.5_ or 6 ppm for CO; and hours above 1,000 μg/m^3^ for PM_2.5_ or 9 ppm for CO. 100 μg/m^3^ is four times the WHO guideline for 24-hour average PM_2.5_ exposure and was used as a threshold in previous study on the association with acute lower respiratory infection [18]. 9 ppm is the exposure threshold above which carboxyhemoglobin level is expected to exceed 2% for a normal subject engaging in light or moderate exercise for 8 hours, while 6 ppm is recommended to address impact of chronic exposure for 24 hours [4].

To further understand air pollutants and concentrations attributed to cookstove usage, an algorithm was developed to differentiate SI and non-stove-influenced (non-SI) periods. Since stove related cooking events would elevate the level of PM_2.5_ and CO, we first defined a baseline concentration and threshold above which measurements were candidates to be defined as SI. Since activities like sweeping and smoking could also increase pollution concentrations for short periods, a filtered time series was obtained for each home by applying a running median smoother of length n to the 5-minute average values to eliminate short peaks. The baseline level was then defined as the *α* (e.g. 10^th^) percentile of this filtered series. This baseline was meant to represent a typical value during the non-SI period. The SI threshold was then defined as a value *β* times the baseline level. The 5-minute intervals for which the filtered values exceeded this threshold were defined as SI, and all other times were defined as non-SI. With this definition of SI, the original 5-minute aggregated data (before the running median smoother of length n) were then used in all subsequent calculations.

The SI partition depends on three constants: n, *α*, and *β*. We studied the dependence of the final daily average concentration measurements on the choice of these constants among the values: n = 5, 7 and 9; *α =* 10, 20 and 30%, and *β* = 1.2, 2.0, and 4.0, producing 27 different average values, one for each combination of constants to study the effect of constant choice on the characterization of SI concentration. The correlation was estimated using the Pearson correlation coefficient for each pair of the 27 averages to determine the influence of the constants on the average SI concentrations.

We also estimated the correlation between daily average PM_2.5_ and CO under different fuel types and seasons using Pearson’s correlation coefficients. The association between pollution concentrations and household characteristics was initially assessed by stratification. Logarithm-transformed concentrations were linearly regressed on household characteristics controlling for fuel type, season, roof material, kitchen wall material, kitchen size and presence of external openings in kitchen. Both daily average PM_2.5_ and CO concentrations among all households had a more nearly symmetric distribution on the logarithmic scale. Logarithm-transformed values were therefore used in all regression models.

## Results

### Household Characteristics

Applying the inclusion criteria described above, the number of daily households with pre-stove installation data available for analysis was 2,980 for PM_2.5_ and 2,013 for CO measurements. The average sampling period per household was 21.7 h (interquartile range: 20.9 h– 22.1 h). In Table 1, we summarized the distributions of household characteristics and season of assessment. More than 70% of houses had walls made from bamboo with mud plaster or wood; the vast majority of roofs were tile or tin. Nearly a third of kitchens were internal rooms with no window or door opening to the outdoors. More than half of households burned wood alone or in combination with crop waste or dung and most of the measurements were conducted during the dry season. Households with missing information on characteristics or environmental factors had similar PM_2.5_ and CO concentrations as those with such data.

**Table 1:** Summary of pre-intervention household characteristics and environmental factors (N = 2980).

Household characteristics	N (%*)
**Fuel type**	
Wood	1175 (39)
Dung/wood	521 (17)
Crop waste	881 (30)
Wood/dung/crop waste	403 (14)
**Season**	
Rainy season (June-September)	893 (30)
Dry season (October-May)	2087 (70)
**Presence of external opening in kitchen**	
Internal room without external window/door	801 (32)
At least one external window/door	1674 (68)
**Roof material**	
Cement	164 (6)
Tile or tin	2509 (84)
Plastic, thatch or grass	305 (10)
**Kitchen wall material**	
Brick or stone with mortar	723 (25)
Bamboo with mud plaster or wood planks	2101 (72)
Thatch, grass, sticks, branches without mud	73 (3)
	(median, interquartile range)
**Kitchen size in m**^**3**^	24.2 (13.6, 35.7)

*-Percentage among households with non-missing household characteristics.

### General description of HAP

In Table 2, we summarized PM_2.5_ and CO concentrations in four metrics. The arithmetic mean and median for daily average PM_2.5_ concentration exceeded 1,000 μg/m^3^, while that for CO concentration were over 8 ppm. The mean and median for the 95^th^ percentile PM_2.5_ concentration were higher than 4,500 μg/m^3^, while that for CO concentration were over 35 ppm. And half of the households in these communities had approximately 15 hours of PM_2.5_ concentrations over 100 μg/m^3^, and 5 hours of that over 1000 μg/m^3^. 15% of households experienced CO levels higher than 9 ppm for more than 8 hours (data not shown).

**Table 2:** Summary of pre-intervention HAP concentrations (N = 2980 for PM2.5; N = 2013 for CO).

	Mean (95% CI)	Median (IQR)
**Overall** PM_2.5_ **Conc. (μg/m**^**3**^**)**		
**Daily Average**	1376 (1331, 1422)	1070 (601, 1757)
**95th percentile**	6423 (6180, 6667)	4817 (2728, 7860)
**Time above 100μg/m**^**3**^ **(h)**	14.3 (14.1, 14.5)	14.8 (8.7, 20.4)
**Time above 1000μg/m**^**3**^ **(h)**	5.3 (5.1, 5.4)	4.8 (2.9, 7.0)
**Overall CO Conc. (ppm)**		
**Daily Average**	10.9 (10.5, 11.3)	8.1 (4.6, 14.5)
**95th percentile**	49.9 (48.0, 51.9)	35.8 (16.0, 70.0)
**Time above 6ppm (h)**	6.6 (6.4, 6.8)	5.8 (3.7, 8.4)
**Time above 9ppm (h)**	4.9 (4.7, 5.0)	4.4 (2.5, 6.6)

**Fig 1:**
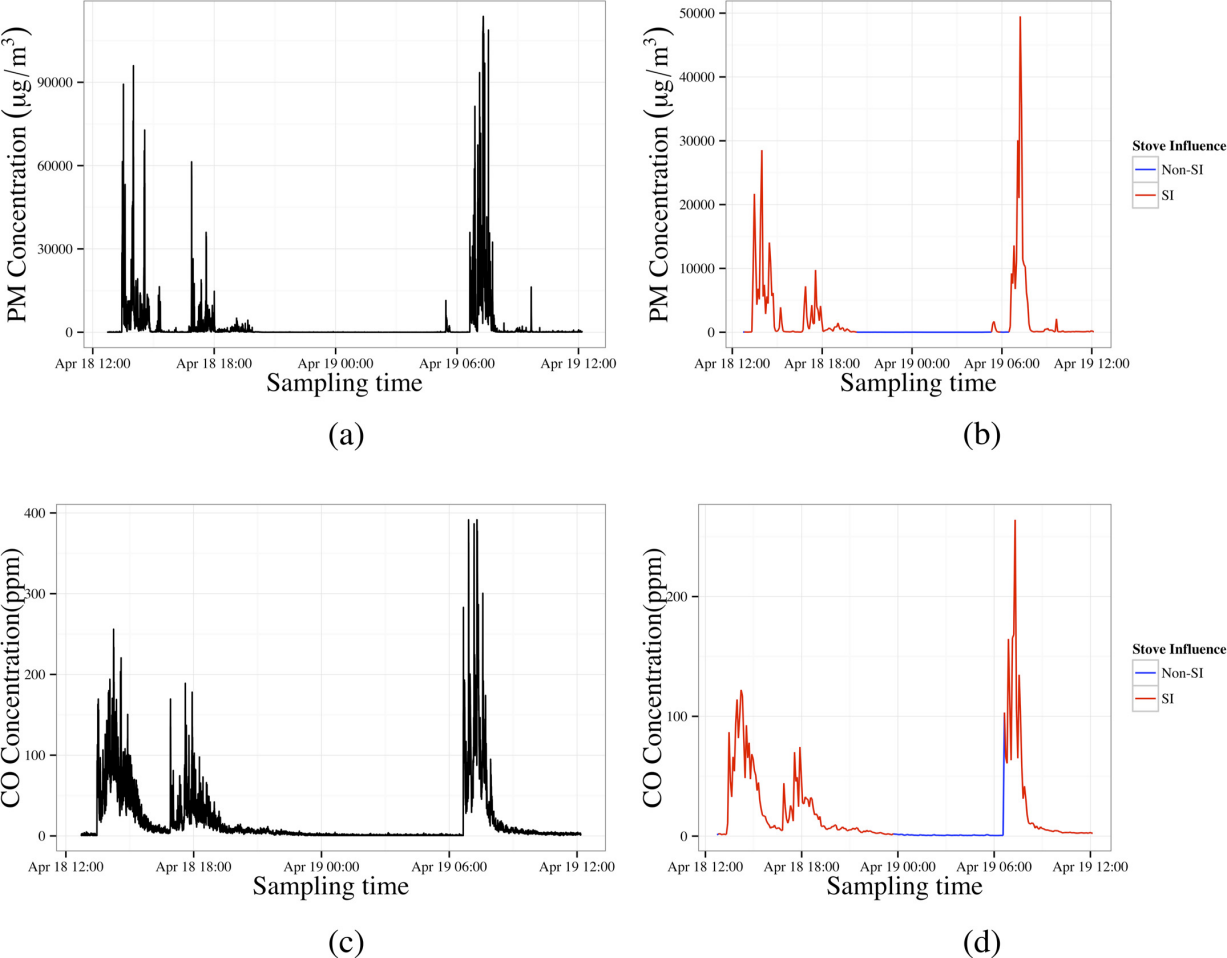
Comparison of PM2.5/CO concentration-time relationship before and after filtering: left, original 10-second interval data; right, aggregated 5-minute interval data with SI period indicated by color (red-SI; blue-non-SI).

Correlations among SI concentrations calculated with different sets of constants were high for both pollutants, ranging from 0.92 to 1.00 for PM_2.5_, and 0.83 to 1.00 for CO. PM_2.5_ results were reported for running medians of length 7 (n = 7), baseline level at the 30^th^ percentile (*α =* 30%), and SI defined to be at levels 4 times the baseline (*β* = 4.0). The corresponding values for CO were chosen as n = 7, *α =* 20%, and *β* = 2.0. Qualitatively similar results are obtained for the other parameter values and are available from the authors. An example of the raw and filtered, then averaged PM_2.5_ and CO data with SI periods highlighted is shown in Fig 1.

In Table 3, we estimated HAP concentrations during SI and non-SI periods. The SI concentrations were about half of the 95^th^ percentile concentrations for both PM_2.5_ and CO, and about 20 times of non-SI concentrations. The non-SI CO concentrations were close to zero while the non-SI PM_2.5_ concentrations were over 100 μg/m^3^.

**Table 3:** Summary of pre-intervention household SI and non-SI HAP concentrations (N = 2980 for PM_2.5_; N = 2013 for CO).

	Mean (95% CI)	Median (IQR)
**PM**_**2.5**_ **Metrics**		
SI Conc. (μg/m^3^)	3469 (3350, 3588)	2700 (1408, 4488)
Non-SI Conc. (μg/m^3^)	268 (252, 284)	139 (19, 399)
SI Time (h)	8.1 (8.0, 8.2)	7.8 (5.8, 10.4)
SI Time (%)	37 (37, 38)	36 (27, 48)
**CO Metrics**		
SI Conc. (ppm)	21.8 (21.1, 22.6)	16.7 (8.9, 29.4)
Non-SI Conc. (ppm)	1.9 (1.8, 2.0)	1.1 (0.6, 2.2)
SI Time (h)	9.7 (9.6, 9.8)	10.0 (7.4, 12.3)
SI Time (%)	45 (44, 46)	46 (34, 57)

**Fig 2:**
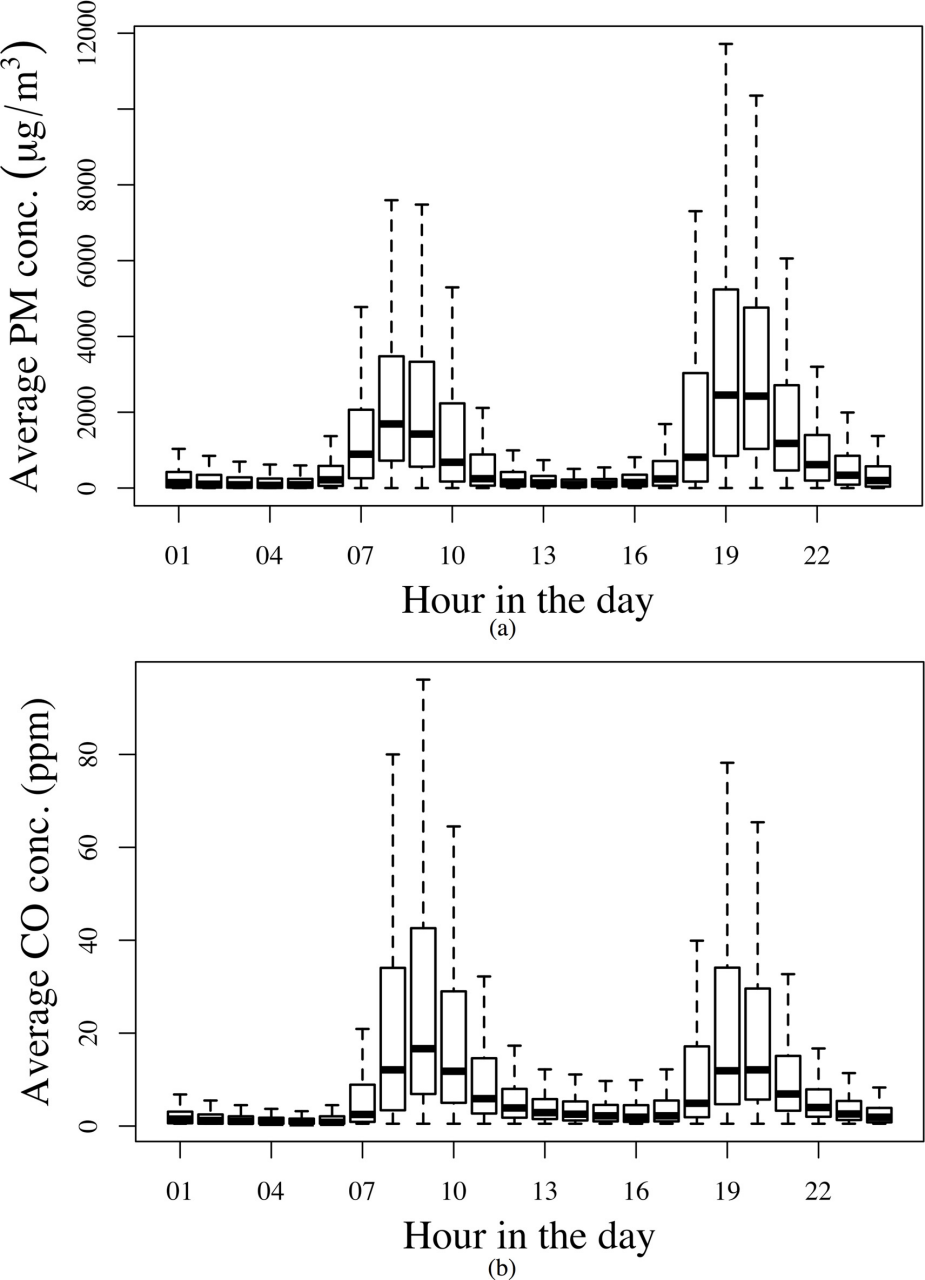
Tukey boxplots of diurnal variation of hourly average (a) PM2.5 concentrations; (b) CO concentrations.

**Fig 3:**
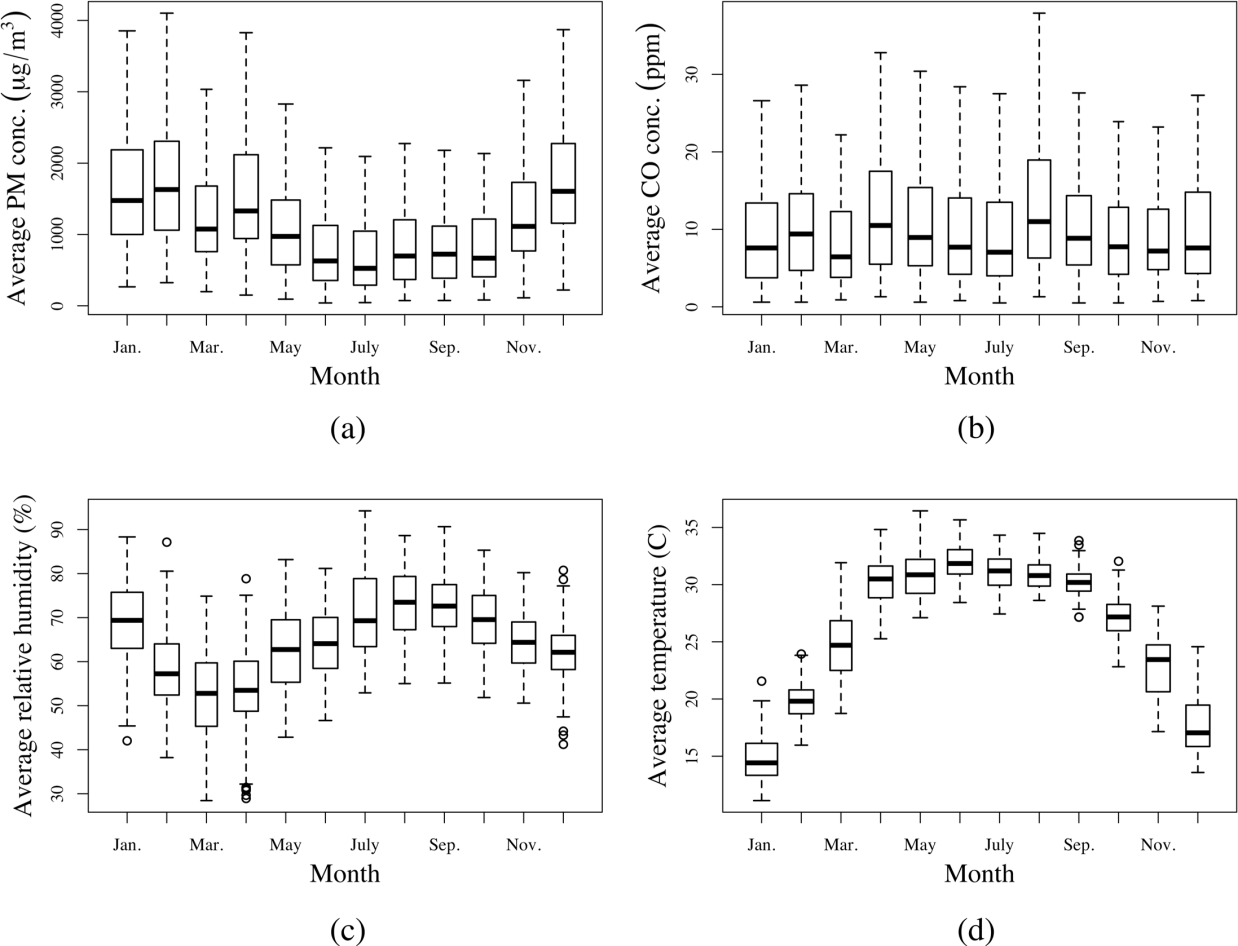
Tukey boxplots of monthly variation of: (a) daily average PM2.5 concentrations*; (b) daily average CO concentrations*; (c) daily average indoor relative humidity; (d) daily average temperature.

Fig 2 presents hourly average PM_2.5_ and CO concentrations. Both pollutants had two peaks (observed elevation in median pollutant concentrations) corresponding to cooking times between 7:00 am and 11:00 am, and then again between 6:00 pm and 10:00 pm, representing the typical cooking pattern in the study population. Total cooking hours under this pattern were similar to the estimated mean and median SI times in Table 3. As displayed in Fig 3(A), the monthly average PM_2.5_ concentration was lower in the rainy season and higher in dry season. For CO (Fig 3(B)), there was no evidence of substantial seasonal variation. Daily average indoor relative humidity and temperature tracked the seasonal outdoor patterns as shown in Figs 3(C) and 2(D).

### Relationship between PM2.5 concentration and CO concentration

**Fig 4:**
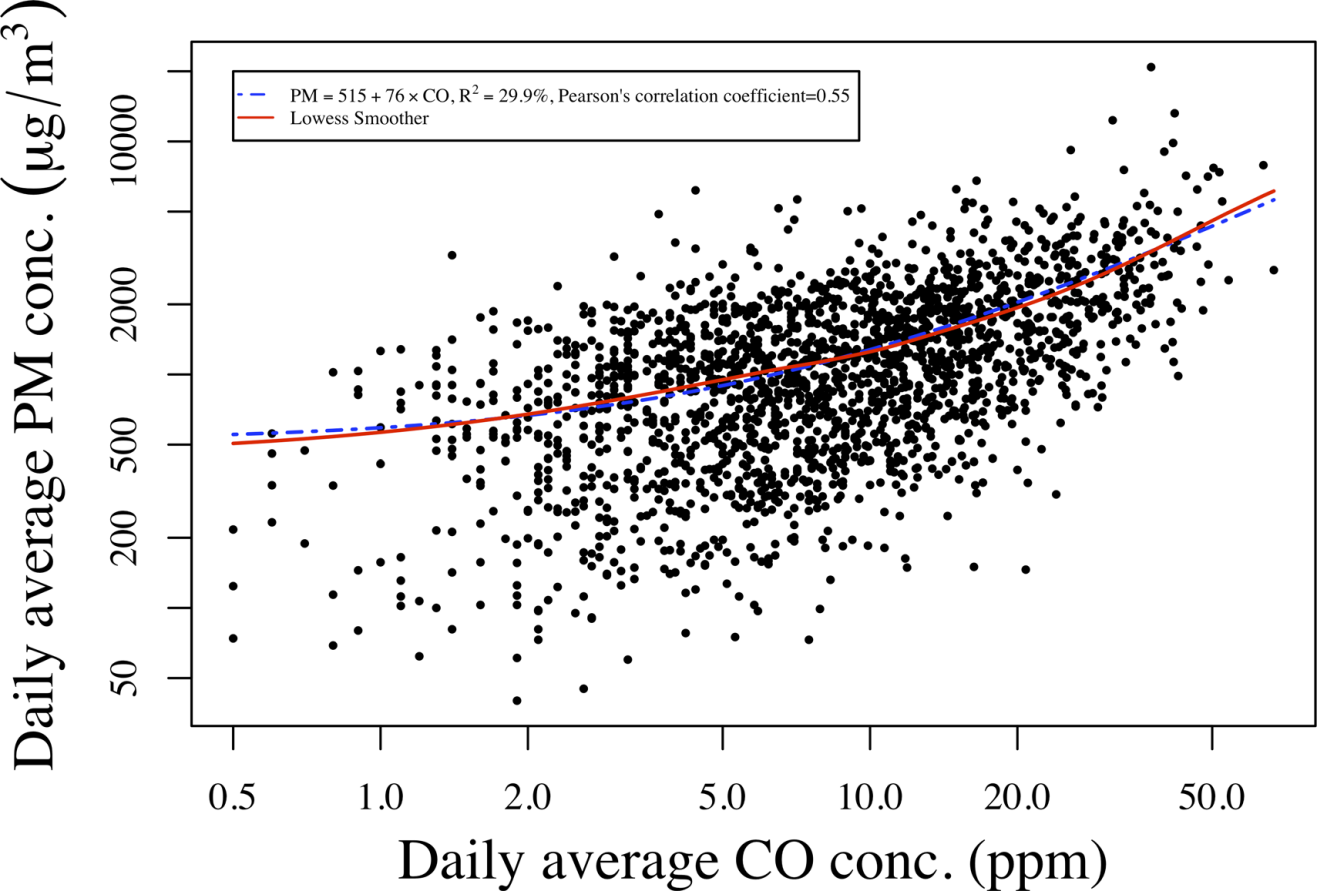
Scatter plot of CO concentration and PM2.5 concentration on logarithmic scales: regression line estimated by simple linear regression and Lowess smoother on original scales.

Fig 4 shows a scatter plot of average log PM_2.5_ against average log CO concentrations with a Lowess curve [19] to estimate the possible non-linear relationship. Log scales were used because each variable had a more nearly symmetric distribution on the log scale. Also displayed was the fitted linear regression of PM_2.5_ against CO, which appeared curvilinear on the log scale. The similarity of the linear regression with the nonparametric Lowess indicates that the PM_2.5_—CO relationship is approximately linear on the original scale. The coefficient of determination for this linear regression is estimated to be 0.30, and the PM_2.5_—CO correlation is estimated to be 0.55 (95% CI: 0.52–0.58). Stratification by season did not improve the correlation, while stratification by fuel type improved the correlations by 20% for dung/wood and dung/wood/crop waste.

### Relationship between HAP and household characteristics

PM_2.5_ concentrations varied across household characteristics even after stratification by season, and the same held for CO concentrations (Tables 4 and 5). PM_2.5_ concentrations in the rainy season were roughly half the corresponding concentrations in the dry season, while CO concentrations remained constant across the year. Among all metrics, non-SI in the rainy season was the only one that yielded PM_2.5_ lower than the WHO 24-hour guidelines, while non-SI in dry season was still 10 times higher than the WHO 24-hour guidelines. Wood burning produced the lowest PM_2.5_ concentrations across the year, while dung/wood was associated with lowest CO concentrations in rainy season; crop waste burning was lowest in the dry season. Presence of an external opening in the kitchen was associated with reduced PM_2.5_ and CO concentrations in the dry season but higher levels in the rainy season. Bamboo with mud plaster or wood planks had the lowest PM_2.5_ and CO concentrations among all kitchen wall materials.

**Table 4:** Summary of pre-intervention PM_2.5_ concentrations stratified by household characteristics in dry season (N = 2980).

	N (%)	Median overall PM_2.5_ conc. (IQR) (μg/m^3^)	Median SI PM_2.5_ conc. (IQR) (μg/m^3^)	Median non-SI PM_2.5_ conc. (IQR) (μg/m^3^)
**Rainy season**				
**Fuel type**				
Wood	331 (11)	522 (316, 963)	1127 (620, 2211)	10 (2, 50)
Dung/wood	247 (8)	697 (371, 1162)	1505 (778, 2526)	13 (3, 72)
Crop waste	167 (6)	718 (391, 1290)	1612 (953, 2767)	14 (3, 57)
Wood/dung/crop waste	148 (5)	753 (436, 1269)	1821 (841, 3489)	24 (5, 102)
**Presence of external opening**				
No	229 (9)	579 (340, 987)	1292 (729, 2396)	12 (2, 67)
Yes	542 (22)	664 (348, 1132)	1481 (706, 2673)	14 (3, 70)
**Roof material**				
Cement	62 (2)	691 (394, 1117)	1457 (898, 2640)	14 (3, 60)
Tile or tin	731 (25)	629 (343, 1089)	1385 (707, 2615)	13(3, 72)
Plastic, thatch or grass	100 (3)	810 (382, 1413)	1708 (942, 3105)	17 (4, 53)
**Kitchen wall material**				
Brick or stone with mortar	228 (8)	769 (432, 1417)	1783 (1031, 3241)	19 (3, 86)
Bamboo with mud plaster or wood planks	631 (22)	590 (333, 1090)	1358 (655, 2538)	12 (3, 62)
Thatch, grass or branches without mud	14 (0)	753 (436, 943)	1507 (1233, 2222)	60 (11, 132)
**Dry season**				
**Fuel type**				
Wood	844 (28)	1186 (710, 1920)	3074 (1791, 4897)	250 (82, 485)
Dung/wood	274 (9)	1371 (878, 2094)	3515 (2248, 5283)	258 (110, 486)
Crop waste	714 (24)	1264 (835, 1900)	3382 (2155, 5298)	308 (122, 548)
Wood/dung/crop waste	255 (9)	1285 (835, 2023)	3370 (1962, 5285)	178 (61, 428)
**Presence of external opening**				
No	572 (23)	1367 (894, 2094)	3567 (2318, 5425)	314 (116, 575)
Yes	1142 (46)	1199 (758, 1846)	3150 (1859, 4823)	246 (90, 468)
**Roof material**				
Cement	102 (3)	1240 (810, 1971)	3190 (1976, 4905)	315 (93, 560)
Tile or tin	1778 (60)	1250 (798, 1958)	3304 (1959, 5075)	258 (91, 493)
Plastic, thatch or grass	205 (7)	1223 (791, 1924)	3319 (1996, 5481)	301 (116, 521)
**Kitchen wall material**				
Brick or stone with mortar	495 (17)	1251 (827, 1981)	3337 (1892, 5223)	258 (68, 532)
Bamboo with mud plaster or wood planks	1470 (51)	1231 (787, 1945)	3282 (1965, 5058)	266 (103, 492)
Thatch, grass or branches without mud	59 (2)	1473 (807, 2097)	3679 (1951, 6389)	167 (51, 486)

**Table 5:** Summary of pre-intervention CO concentrations stratified by household characteristics in rainy season (N = 2013).

	N (%)	Median overall CO conc. (IQR) (ppm)	Median SI CO conc. (IQR) (ppm)	Median non-SI CO conc. (IQR) (ppm)
**Rainy season**				
**Fuel type**				
Wood	257 (13)	8.8 (4.8, 16.2)	16.8 (9.5, 32.2)	1.6 (0.7, 3.9)
Dung/wood	196 (10)	8.2 (5.2, 14.6)	17.0 (9.0, 28.7)	1.7 (0.7, 3.9)
Crop waste	118 (6)	8.7 (4.3, 15.6)	19.1 (8.6, 34.9)	1.7 (0.7, 2.9)
Wood/dung/crop waste	105 (5)	8.9 (5.0, 14.3)	20.0 (11.6, 34.3)	1.8 (0.8, 3.0)
**Presence of external opening**				
No	170 (10)	8.3 (4.5, 15.1)	17.1 (9.0, 30.1)	1.6 (0.7, 3.8)
Yes	414 (25)	8.5 (5.0, 15.4)	18.3 (9.5, 32.4)	1.7 (0.6, 3.1)
**Roof material**				
Cement	45 (2)	8.9 (5.6, 13.4)	16.3 (10.5, 30.3)	1.5 (0.7, 2.9)
Tile or tin	559 (28)	8.1 (4.7, 14.8)	16.8 (9.2, 30.0)	1.7 (0.7, 3.4)
Plastic, thatch or grass	72 (4)	12.3 (5.8, 19.4)	26.7 (15.3, 42.1)	1.8 (0.7, 4.2)
**Kitchen wall material**				
Brick or stone with mortar	176 (9)	9.2 (5.7, 16.5)	20.2 (11.6, 34.6)	1.7 (0.7, 2.7)
Bamboo with mud plaster or wood planks	472 (24)	7.9 (4.6, 15.0)	16.9 (9.1, 31.0)	1.7 (0.7, 3.6)
Thatch, grass or branches without mud	11 (1)	8.9 (4.9, 16.2)	16.2 (8.7, 34.1)	2.1 (1.7, 4.3)
**Dry season**				
**Fuel type**				
Wood	544 (27)	8.2 (4.6, 14.5)	16.6 (8.7, 27.7)	0.8 (0.6, 1.7)
Dung/wood	173 (9)	8.8 (5.0, 16.8)	16.9 (9.6, 35.0)	1.1 (0.6, 2.2)
Crop waste	463 (23)	6.8 (3.9, 12.8)	14.6 (7.6, 26.9)	0.7 (0.6, 1.5)
Wood/dung/crop waste	157 (8)	9.3 (5.7, 13.4)	18.5 (11.0, 29.6)	1.2 (0.6, 2.2)
**Presence of external opening**				
No	377 (22)	8.0 (4.7, 14.2)	15.9 (9.5, 27.0)	0.7 (0.6, 1.6)
Yes	716 (43)	7.5 (4.1, 13.5)	15.6 (8.1, 27.1)	0.8 (0.6, 1.9)
**Roof material**				
Cement	57 (3)	7.3 (4.5, 17.4)	14.3 (7.1, 31.6)	1.0 (0.6, 2.7)
Tile or tin	1145 (57)	7.9 (4.3, 14.1)	15.9 (8.5, 28.0)	0.8 (0.6, 1.8)
Plastic, thatch or grass	134 (7)	9.3 (4.9, 14.6)	19.7 (10.3, 28.0)	0.7 (0.6, 1.7)
**Kitchen wall material**				
Brick or stone with mortar	314 (16)	9.4 (5.3, 15.1)	18.0 (10.4, 31.1)	1.0 (0.6, 2.0)
Bamboo with mud plaster or wood planks	939 (48)	7.6 (4.1, 13.6)	15.4 (8.0, 26.8)	0.8 (0.6, 1.6)
Thatch, grass or branches without mud	39 (2)	8.1 (5.1, 20.9)	17.4 (10.7, 40.9)	1.8 (0.6, 2.7)

Table 6 presents the results of models in which the average daily concentrations were regressed on season and household characteristics. Higher PM_2.5_ concentrations were associated with fuels other than wood, dry season, having an internal kitchen without an external window/door, larger kitchen size, and wall material other than bamboo with mud plaster or wood planks. Higher values of CO concentrations were associated with fuels other than crop waste, rainy season, smaller kitchen size, and wall material other than bamboo with mud plaster or wood planks (Table 7).

**Table 6:** Relative and absolute differences in PM2.5 concentrations predicted by household characteristics and season.

	Unit of increase	Overall PM_2.5_(95% CI)	SI-PM_2.5_(95% CI)	Non-SI-PM_2.5_ (95% CI)
**Intercept**	μg/m^3^	678 (583, 788)	1482 (1265, 1737)	22 (16, 30)
**Fuel type (ref. Wood)**				
Wood/Dung	**%**	**20 (10, 32)**	**21 (10, 33)**	**29 (7**, **57)**
	μg/m^3^	**138 (69, 214)**	**309 (150, 485)**	**6 (1, 12)**
Crop waste	**%**	**12 (4, 21)**	**19 (9, 29)**	**27 (8**, **50)**
	μg/m^3^	**79 (24, 140)**	**276 (140, 423)**	**6 (2, 11)**
Dung/Wood/Crop waste	**%**	**17 (6, 29)**	**24 (12, 37)**	5 (-15, 30)
	μg/m^3^	**113 (40, 195)**	**351 (172, 549)**	1 (-3, 7)
**Dry season (ref. Rainy season)**	**%**	**99 (85, 113)**	**127 (111, 144)**	**922 (781**, **1085)**
	μg/m^3^	**668 (579, 763)**	**1880 (1647, 2131)**	**202 (172, 238)**
**Presence of external opening in kitchen (ref. No external opening in kitchen)**	**%**	**-10 (-16, -3)**	**-10 (-17, -3)**	**-24 (-35**, **-12)**
	μg/m^3^	**-68 (-109, -24)**	**-156 (-246, -52)**	**-5 (-8, -3)**
**Roof (ref. Cement)**				
Tile or tin	**%**	-1 (-13, 13)	-1 (-14, 14)	-7 (-30, 23)
	μg/m^3^	-7 (-90, 88)	-9 (-201, 210)	-2 (-7, 5)
Plastic, thatch or grass	**%**	14 (-4, 34)	17 (-2, 39)	10 (-23, 57)
	μg/m^3^	94 (-24, 233)	253 (-24, 583)	2 (-5, 13)
**Wall (ref. Birck or stone with mortar)**				
Bamboo with mud plaster or wood planks	**%**	**-15 (-21, -8)**	**-17 (-23, -10)**	**-17 (-29, -2)**
	μg/m^3^	**-102 (-143, -57)**	**-245 (-339, -143)**	**-4 (-6, 0)**
Thatch, grass or branches without mud	**%**	-13 (-29, 7)	-13 (-29, 8)	-32 (-56, 5)
	μg/m^3^	-86 (-195, 47)	-186 (-435, 121)	-7 (-12, 1)
**Kitchen size in 10 m**^**3**^ **(ref. median)**	**%**	0 (-1, 2)	1 (-1, 3)	**4 (1, 8)**
	μg/m^3^	3 (-9, 15)	13 (-14, 41)	**1 (0, 2)**

**Table 7:** Relative and absolute differences in CO concentrations predicted by household characteristics and season.

	Unit of increase	Overall CO(95% CI)	SI- CO(95% CI)	Non-SI- CO (95% CI)
**Intercept**	ppm	9.8 (8.0, 12.0)	18.0 (14.8, 21.9)	1.7 (1.4, 2.0)
**Fuel type (ref. Wood)**				
Wood/Dung	**%**	2 (-9, 14)	2 (-9, 14)	8 (-4, 21)
	ppm	0.2 (-0.9, 1.4)	0.3 (-1.7, 2.4)	0.1 (-0.1, 0.4)
Crop waste	**%**	**-13 (-22, -4)**	-5 (-14, 5)	-9 (-17, 1)
	ppm	**-1.3 (-2.1, -0.4)**	-0.9 (-2.5, 0.8)	-0.1 (-0.3, 0)
Dung/Wood/Crop waste	**%**	5 (-8, 19)	13 (0, 28)	12 (-2, 27)
	ppm	0.4 (-0.8, 1.9)	2.3 (-0.1, 5)	0.2 (0, 0.4)
**Dry season (ref. Rainy season)**	**%**	-6 (-14, 2)	**-10 (-18, -2)**	**-33 (-39, -27)**
	ppm	-0.6 (-1.4, 0.2)	**-1.8 (-3.2, -0.4)**	**-0.6 (-0.6, -0.5)**
**Presence of external opening in kitchen (ref. No external opening in kitchen)**	**%**	-2 (-11, 7)	1 (-7, 11)	0 (-9, 9)
	ppm	-0.2 (-1.1, 0.7)	0.2 (-1.3, 1.9)	0 (-0.2, 0.2)
**Roof (ref. Cement)**				
Tile or tin	**%**	1 (-15, 21)	4 (-13, 24)	-4 (-19, 15)
	ppm	0.1 (-1.5, 2.1)	0.7 (-2.3, 4.2)	-0.1 (-0.3, 0.2)
Plastic, thatch or grass	**%**	19 (-4, 49)	**35 (9, 67)**	-5 (-23, 18)
	ppm	1.9 (-0.4, 4.8)	**6.2 (1.6, 12)**	-0.1 (-0.4, 0.3)
**Wall (ref. Birck or stone with mortar)**				
Bamboo with mud plaster or wood planks	**%**	**-20 (-27, -11)**	**-16 (-23, -7)**	0 (-9, 9)
	ppm	**-1.9 (-2.6, -1.1)**	**-2.8 (-4.2, -1.3)**	0 (-0.2, 0.2)
Thatch, grass or branches without mud	**%**	-5 (-26, 24)	0 (-22, 28)	**36 (5, 75)**
	ppm	-0.5 (-2.6, 2.3)	-0.1 (-4.0, 5.0)	**0.6 (0.1, 1.3)**
**Kitchen size in 10 m**^**3**^ **(ref. median)**	**%**	**-3 (-5, -1)**	**-4 (-6, -2)**	1 (-1, 3)
	ppm	**-0.3 (-0.5, -0.1)**	**-0.7 (-1.1, -0.3)**	0 (0, 0.1)

## Discussion

We present a methodology for pre-processing and quantifying average indoor concentrations of PM_2.5_ and CO in a representative sample of nearly 3,000 households in rural southern Nepal. We established criteria for data pre-processing to ensure the quality of data and efficiency of analysis. From every 10 seconds sampling, we used non-linear filters to eliminate spurious outliers, averaged the resulting values into 5-minute interval data that were used to estimate SI and non-SI periods. SI and non-SI concentrations were then used to characterize HAP.

The method for estimating SI and non-SI includes 3 constants that can be used to tune the methods for this or other applications. The first is the length of a running median that eliminates shorter excursions that are more likely caused by suspension of settled particles through sweeping or other indoor activities rather than lighting of the stove. The second establishes the quantile of the 5-minute data series that should be used as the baseline level. This controls the length of time that could potentially be categorized as SI and represents a typical value during the non-SI period. The third constant defines a threshold that is a multiple of the typical non-SI concentration, allowing fluctuation in non-SI period. Since this study was conducted in an extremely poor environment with a per capita income of $146 [20], the cookstove is used for both cooking and heating, which makes it the main source of indoor air pollutants. It is reasonable to assume that these relatively long peaks identified were mostly caused by cookstove related activities. When deciding on the actual number of 3 constants, we compared results from different groups of constants to avoid misclassification of smaller peaks from fire star-up and end of burn periods.

We also differentiated SI and non-SI exposures by identifying peaks through the change of differences in value between two neighboring data points, and setting rules to combine or exclude identified peaks [21]. It requires two constants to establish change qualified for peak, one to establish the length for combining peaks, and the other to establish the length for excluding peaks. This method is more intuitive, but also more sensitive to change in constants, and requires more intensive adjustment in achieving an acceptable result. The results from this method had a correlation coefficient of 0.85 for PM_2.5_ and 0.87 for CO with results from the first method. Although misclassification exists both methods, we would recommend the first method given it is easier to generalize.

This study revealed extremely high HAP concentration with traditional biomass cookstoves in rural areas of southern Nepal. It demonstrated patterns with both high concentration peaks and long periods of elevated concentrations. Average PM_2.5_ concentrations for daily, SI period, and non-SI period were 40, 100, and 10 times higher than the WHO guidelines. The use of a passive device for measuring PM_2.5_ concentrations is subject to error when humidity levels are high and when air currents around the device are irregular. To address this concern, we calibrated this passive device against a gold standard gravimetric approach [14]. All concentrations reported herein are adjusted by this calibration. The calibration study was conducted in both a model house and local participating houses typical of the study setting. We did not limit movement of persons in the houses and, thus, some error could have been induced in our measurements from irregular air currents passing through the detection chamber of the device. While we are unable to estimate the level of this potential error in measurement, for the purposes of the randomized trial, there is no reason to expect this potential error would be different before and after installation of the improved biomass stove.

It is clear that controlling of SI PM_2.5_ could significantly reduce the indoor concentration levels. The estimated high non-SI PM_2.5_ concentrations could be a result of elevated community baseline PM_2.5_ concentrations due to SI PM_2.5_ exfiltration from other households or a contribution from other sources such as road dust, suggesting that community wide interventions may be necessary for reducing PM_2.5_ concentrations to a significant degree. In contrast, compared with WHO guidelines, average CO concentrations for daily and SI periods was only 37% and 3 times higher respectively, while the CO concentration for non-SI periods was close to zero, indicating that successful control of SI CO could reduce the overall concentrations to an acceptable level. Given the fact that most households were sampled between 3pm and 12pm for logistic reasons, and that local people do not cook around noon, the daily HAP reported here might be higher than the actual 24-hour time weighted HAP in these households. The same situation might exist for non-SI concentrations but not for SI concentrations.

Since few data were available on daily HAP related to biomass fuel combustion with traditional cookstoves in rural Nepal, comparisons were made with studies carried out in rural India, where similar types of cooking behaviors, fuels, traditional stoves, and geographic and climate characteristics are present. Previous studies reported daily average PM_2.5_ concentrations ranging from 686 to 1,250 μg/m^3^ and daily average CO concentrations ranging from 2.6 to 10.8 ppm, both similar but lower than 1,377 μg/m^3^ and 10.9 ppm reported in our study [10,11]. Previous studies in rural Nepal also identified 4,741 μg/m^3^ PM_2.5_ concentrations and 13.7 ppm CO concentrations during cooking periods, which are also close to 3,466 μg/m^3^ and 21.9 ppm reported in our study [8,9].

Season was identified as the most significant influential factor in PM_2.5_ concentrations, especially in the non-SI period. Given the relatively low temperature in dry season, changing behaviors such as closing windows or doors, and prolonged periods of fuel burning as a source of heat could explain the increase in overall and SI PM_2.5_ concentrations. Increases in non-SI PM_2.5_ concentrations could result from the lack of a dampening effect of rain on ambient particulate matter, and increases in background PM_2.5_ concentrations in non-SI period due to closed windows and doors. However, CO concentrations were slightly increased in the rainy season, potentially caused by more inefficient combustion with wet fuel. Having an external opening to the outdoors in the kitchen also decreased the indoor PM_2.5_ concentration, while CO was not impacted as much. The effect of external openings should be interpreted with caution because the status of these openings was not recorded during sampling. Wood was the cleanest fuel for both pollutants, while adding dung into the fuel led to worse indoor air quality. Other household characteristics such as the type of roof and wall material did not have consistent associations with concentrations of HAP.

A previous study from Guatemala suggested the use of CO as a surrogate for PM_2.5_ [22] based on high correlations between these two pollutants [23]. However, the overall correlation between PM_2.5_ and CO in this study was 0.55, lower than reported in Guatemala [22,23]. The coefficient of determination for this linear regression is estimated to be 0.30, lower than previously reported 0.73 to 0.78 in Guatemala [24]. The increases in correlation through stratification were also lower than a 2 fold increase reported from China [25]. This suggests that the use of CO concentration as a surrogate for PM_2.5_ concentration is less appropriate on our setting, even after stratification by fuel type and season.

## Conclusions

In this paper, we summarized the time series pre-processing in estimating daily average concentrations from the original 10-second intervals data. A simple, flexible method developed to distinguish periods during which pollution concentrations are SI or non-SI improved the understanding of HAP and could be easily applied to other studies by tuning 3 constants in the algorithm.

We also filled a gap in knowledge on HAP in rural Nepal with daily concentration data collected in ~3,000 households and revealed the severity of the HAP problem in rural Nepal. For households utilizing a traditional open burning mud stove, the median daily average PM_2.5_ concentration was over 40 times higher than the WHO guideline for daily exposure, and the median daily average CO concentration was about 30% higher than the WHO recommended guideline for daily exposure. A detailed description of the concentrations using multiple metrics will also facilitate further analysis on health outcomes for NCIT-I that will be reported in a future manuscript. Exploration into the influence of environmental factors and household characteristics on HAP provided potential intervention methods for reducing indoor air pollution.

## Supporting Information

S1 File Data for Fig 1 10 second intervals. (CSV)

S2 File Data for Fig 1 5 minute intervals. (CSV)

S3 File Data for Fig 2. (CSV)

S4 File Baseline data file. (CSV)

S5 File Baseline data file codebook. (XLSX)
